# Palliative sedation for terminally ill cancer patients in a tertiary cancer center in Shanghai, China

**DOI:** 10.1186/s12904-015-0002-6

**Published:** 2015-03-15

**Authors:** Xiaoli Gu, Wenwu Cheng, Menglei Chen, Minghui Liu, Zhe Zhang

**Affiliations:** Department of Integrated Therapy, Fudan University Shanghai Cancer Center, #270, Dong’An Road, Shanghai, 200032 People’s Republic of China; Department of Oncology, Shanghai Medical College, Fudan University, #270, Dong’An Road, Shanghai, 200032 China

**Keywords:** Palliative sedation, Terminally ill cancer patients, Survival, End of life, Symptom management, Agitated delirium

## Abstract

**Background:**

There are a number of studies dedicated to characteristics of sedation, but these studies are mostly bound to western country practices. The aim of this study is to describe the characteristics of patients who suffered from cancer and who had been sedated until their death in Shanghai, China.

**Methods:**

Retrospective medical data of 244 terminally ill cancer patients including 82 sedated patients were collected. Data collected included demographic characteristics, disease-related characteristics and details of the sedation.

**Results:**

In sedated cases, patients and/or caregivers gave the consent to start palliative sedation due to unmanageable symptoms. On average, sedation was performed 24.65(±1.78)hours before death. Agitated delirium and dyspnea were the most frequent indications for palliative sedation. There was no significant difference in survival time from admission till death between sedated and non-sedated patients (p > 0.05).

**Conclusions:**

Palliative sedation is effective for reducing terminally ill cancer patients’ suffering without hastening death. Prospective research is needed to determine the optimal conditions for Chinese patients including indications, decision making process, informed consent, cultural and ethical issues, type of sedation and drugs.

## Background

### Mini abstract

Palliative sedation is effective for reducing the suffering of terminally ill cancer patients without hastening death. Agitated delirium and dyspnea were the most frequent indications for palliative sedation.

Terminally ill cancer patients may suffer severe refractory symptoms in their last weeks of life despite having received palliative treatments. Palliative sedation (PS) is one of the optimal palliative therapies used for these patients who are resistant to any other forms of treatments [1,2]. According to the definition proposed by the National Hospice and Palliative Care Organization (NHPCO), PS is the lowering of patients’ consciousness using medications for the express purpose of limiting patients’ awareness of suffering that is intractable and intolerable, or sufferings that patients perceive to be unbearable, which has not adequately respond to any interventions and for which additional interventions are either unavailable or impractical [3]. PS can be performed intermittently or continuously until death, and that the depth of sedation can vary from a lower level of consciousness to complete unconsciousness.

PS is frequently used in end-of-life care for terminally ill cancer patients in Western countries. Guidelines and recommendations have been published by different organizations according to different cultural backgrounds and practices such as NHPCO, European Association for Palliative Care(EAPC), the Royal Dutch Medical Association [4-6]. Several inconsistencies exist amongst the guidelines with regards to the initiation, the level, the pattern and even the continuation or discontinuation of life-sustaining therapies. These discrepancies can be attributed to divergent PS definitions, ethical and clinical factors in different areas [7,8].

China is a traditional East Asian country with a unique view on end-of-life issues [9]. Traditional Chinese culture dictates that a good death can only be obtained if consciousness is kept clear near the end of life. For Chinese patients and their families, they may be concerned about the doctrine of double effects of PS. PS may also be confused with euthanasia and physician-assisted suicide because of its potential life-shortening effect. Therefore, it is challenging to follow the western recommendations for palliative care while taking into account the differences in Chinese culture.

There are currently no guidelines or any other rules for PS for Chinese cancer patients. There are not even any formal studies of PS for terminally ill cancer patients. Thus, we present an analysis of PS practice from a Tertiary Metropolitan Cancer Center in Shanghai, China. This study aims to summarize the characteristics of patients who were sedated for refractory symptoms and describe and reflect on some different practices in Mainland China.

## Methods

### Palliative sedation policy and decision making process in the department

Fudan University Shanghai Cancer Center (FUSCC) is an 800-bed tertiary cancer center. The Integrated Therapy Department (used called Palliative Care Department) with a 12-bed inpatient ward was established in 2006 to provide supportive care for advanced cancer patients who cannot undergo any anti-cancer therapy. In this department, daily multidisciplinary meetings are held with palliative care physicians, medical oncologists, nurses, and weekly meetings with an anesthesiologist, a psychiatrist, and social workers.

The sedation was performed according to the sedation routine of our department, which was established through previous experience and clinical research [10], and is described below. PS was potentially indicated for patients with refractory physical symptoms. Initially, intermittent sedation was used. The decision to transition from intermittent to continuous sedation for these patients was based on the prevalence of uncontrolled symptoms. Once PS was implemented, the patients’ vital signs were monitored by nurses regularly during the initial 24 hours. Cessation of sedation was considered when the symptoms became manageable. The indications to start PS, the drugs used for and the duration of the sedation, the decision-making process were recorded in patients’ medical records.

Every patient admitted to the in-patient department of the FUSCC has the option to sign a letter of authorization giving family members the right to make medical decisions for him should he lose his decision-making capacities. For patients lacking decisional capacities, permission was obtained from a legal proxy. The decision-making process for PS was undertaken by a multi-professional palliative care team with more than 3 senior attending palliative physicians to discuss whether PS would ease the patients’ sufferings. PS was recommended by a patients’ attending physician according to the patients’ symptom sufferings and estimated survival length time. A written informed consent form was signed when physicians and patients and/or authorized family members reached an agreement to implement PS after the aims, benefits and risks of PS were informed to patients and/or authorized family. After the written informed consent form was signed by the patients and/or authorized family, sedation drugs were prescribed by the patients’ attending physician.

### Patients and measurements

This study was approved by the Institutional Review Board of the Cancer Center. A systematic retrospective analysis was performed of the medical records of those who died in the Integrated Therapy Department in FUSCC between March 2007 and September 2011.

We reviewed the medical records of the 244 patients through the Union Medical System (UMS) of FUSCC. (The UMS is the electronic medical records system which containing all the medical information of patients in the FUSCC.) Three physicians reviewed the medical records of patients to select patients who received PS for the purpose of palliation. Patients who received benzodiazepines (such as estazolam) for insomnia as symptom control were excluded from this research. Our unit did not use PS for management of anxiety or existential suffering alone.

Demographic variables and details of PS of these patients were gathered and reviewed. The demographic variables included sex, age, diagnosis, metastatic sites and functional/performance status at the day of admission as measured by using the Karnofsky Performance Scale (KPS) ranging from 100 (normal) to 0 (dead).The details of sedation records included the indication to start PS, the duration of the PS, the sub-type of PS, drugs and dosage used, and the administration route of PS. The duration of PS was defined as the number of hours elapsed between the start of PS until it was stopped or the patients died. The sub-type of PS was intermittent or continuous sedation. Survival time was defined as the time between the day of admission to the hospital and the day patients died. The total survival time was defined as the time between the day patients were diagnosed and the day patients died.

The informed consent forms were retrieved through the UMS to evaluate the decision-making process of patients and their family caregivers.

### Statistical analysis

Data management and statistical analysis were performed using SPSS 16.0 software. Frequency distributions were used to describe the demographic data and the distribution of each variable. A Chi-squared test was used to make comparisons with respect to categorical variables, and Fisher’s exact test was used if sample size criteria were not met for Chi-squared approximation. The Kaplan-Meier and Log-rank test were used to compare the survival time between sedated and non-sedated patients. A p-value less than 0.05 was considered statistically significant.

## Results

### Demographic and survival differences between sedated and non-sedated patients

General characteristics of enrolled patients are shown in Table 1. 244 terminally ill cancer patients were included in this study. 51.23% were men. The median age was 63 years old (range 24 to 93 years old). Lung cancer was the most common diagnosis (14.8%). 156 of 244 (63.93%) patients had metastasis. Lung, liver and bone were the most prevalent metastatic sites, and 55 patients (22.54%) had more than 3 sites of metastasis. The most frequent KPS was 20, indicating that patients were mainly bedbound and required assistance with care. The mean survival time was 23.54 days (95% CI: 22, 27), the median survival time was 19 days (95% CI: 17, 21), and the range was 1–139 days.

**Table 1:** **Demographic and survival difference between sedated and non**-**sedated patients**

**Variables**	**Total**	**Sedated** **(N = 82)**	**Non**-**sedated** **(N = 162)**	**P value**
Total	244	82	162	-
Mean age (years)	63	62	63	0.86
Gender (male/female)	125/119	40/42	85/77	0.34
KPS (median)	20	20	30	0.12
Primary tumor site (N/%)				0.60
Lung cancer	36	12/14.6%	24/14.8%	
Liver cancer	30	12/14.6%	18/11.1%	
Breast cancer	26	8/9.8%	18/11.1%	
Stomach cancer	33	9/11.0%	24/14.8%	
Colon cancer	22	8/9.8%	14/8.6%	
Nasopharyngeal cancer	6	2/2.4%	4/2.5%	
Kidney/Prostate cancer	17	5/6.1%	12/7.4%	
Pancreas	20	7/8.5%	13/8.0%	
Gallbladder	9	3/3.7%	6/3.7%	
Hematological Cancer	4	2/2.4%	2/1.2%	
Female genital tract cancer	20	8/9.8%	12/7.4%	
Primary unknown	7	2/2.4%	5/3.1%	
Others	12	2/2.4%	10/6.2%	
Survival time (days)(mean) (95CI)*	23.54	27.44 (23.2-31.65)	21.56 (18.52-24.59)	0.066
Total Survival time (months)(mean) (95CI )#	25.86	35.65 (26.55-44.75)	20.90 (16.72-25.10)	0.002

* Survival time means the days since administration till death.

# Total Survival time means the months since diagnose till death.

There were no differences between sedated and non-sedated patients in gender, age, and primary cancer sites. The median KPS of the sedated patients was lower than that of non-sedated patients (20 and 30, respectively), but the difference was not significant (p > 0.05). The similar characteristics of the sedated and non-sedated patients indicated that the survival difference of the two groups could be compared. The sedated patients had a mean survival time of 27.44 days while the non-sedated patients had a mean survival time of 21.56 days. There was no statistically significant difference between the survival time of the two groups (p = 0.066). Sedated patients had a significantly longer total survival time than non-sedated patients (p = 0.002).

### Details of sedation

The details of sedation are shown in Table 2. 54 out of 82 (65.85%) patients began sedation 0–24 hours before death and 18 (21.95%) patients began sedation 25–48 hours before death. On average, sedation began 24.65 hours (SD ± 1.78, range: 2-71hours) before death. The median time was 22 hours. No patients stopped receiving sedation once the sedation had begun.

**Table 2:** **Details of palliative sedation in 82 patients**

**Variables**	**N**
Frequency of palliative sedation (N/%)	
Intermittently	82(100%)
Intermittently to continuously	20
Medication for sedation*	
Diazepam	59
Haloperidol	48
Chlorpromazine	9
Route ※	
Intravenous injection	3
Intramuscular injection	81
Sedation Duration	
Mean hour (SD)	24.65(±1.78)
Median hour (range)	22 (2–71)
Time between the start of sedation and death	
0-24 hours before death (%)	54 (65.85%)
24-48 hours before death (%)	18 (21.95%)
>48 hours before death (%)	10 (12.20%)
Symptom indication for request (%) #	
Agitated Delirum	39
Dyspnea	35
Pain	12
others	9
Consent	
Caregivers consent only	65
Patientss’ consent only	7
Both the caregivers and patients consent	10

* 34 patients used more than one kind of drugs.

※ The number in the route was 84 because 2 patients received both intravenous and intramuscular injection.

#10 patients suffered more than one indication for the PS. Others included uncontrolled nausea and vomiting.

All 82 patients were intermittently sedated at the beginning, allowing minimal contact with relatives. 20 patients transferred from intermittent to continuous sedation between the start of sedation and death. The mean transfer point was 13.45 hours (SD ± 13.5) before death. The most commonly used drugs were diazepam in 59 patients, haloperidol in 48 patients and chlorpromazine in 9 patients. 34 patients used more than one kind of drug.

Diazepam and haloperidol were used for intermittent PS. Chlorpromazine was used for intermittent PS or in combination with other drugs for continuous PS. Morphine was used in combination with other drugs for 12 of the 82 sedated patients. The most frequent route of administration was intramuscular injection.

Agitated delirium and dyspnea were the most frequent indications for starting PS. 39 received sedation for agitated delirium, 35 for dyspnea, and 12 patients for uncontrolled pain.

### Decision making

All 82 patients had signed the letter of authorization at admission. In 65 cases only the caregivers’ consent was received. This was frequently related to cognitive impairment of various etiologies. Communication was no longer possible or was reduced to the most basic issues with these patients. In 7 cases only the patient’s consent was obtained; in 10 cases both the patients’ and caregivers’ consents were received.

## Discussion

This study provided the overview of the characteristics of PS implementation in Mainland China. Our study revealed that 33.6% of the patients who died in the Integrated Therapy Department received some form of PS. The cultural values of each society play an important role in shaping the practice of sedation according to different definitions, cultural understandings and traditions. This is reflected in several dimensions of the details of PS at FUSCC.

There was no statistically significant difference in survival time between patients who underwent sedation and those who did not. This indicated that PS had no potential life-shortening effect in consist with previous researches [11-15]. Sedation patients had a significantly longer total survival time than non-sedated patients in our study. This may be explained by that patients who had a longer disease journey suffered more and had the more desire to reduce the suffering.

In this study, the frequency of sedation was 33.6% (82/244), similar with a review enrolled 10 studies with 1807 patients (34.4%) [2]. But our rate of PS is higher than that reported in a Taiwanese study more than 10 years ago (27.9%) and in a Singaporean study (22.6% at 48 hours before death) in 2012 [16,17]. Although the Taiwanese, Singaporeans and Mainland Chinese share a similar cultural background, the difference still exists. This difference can be explained by the large variability of PS frequency, which was reported as 1-88% according to cultural understanding of PS and different time period examined [18,19].

The median duration of PS in our department was 22 hours, while the median time in previous studies varied from 21.6 to 48 hours [12,20-23]. 65.85% of our sedated patients died within 24 hours. The potential explanation for shorter duration and late initiation maybe was that patients were usually at the stage of impending death when they started to receive PS. From the physicians’ perspective, estimating the patients’ remaining survival time was considered to be a difficult and challenging task. From the patients and caregivers’ perspective, sedation was seen as having a potential life-shortening effect. These reasons may have lead to PS being started too late and patients suffering needlessly at the end of life.”

As found in other studies, agitated delirium and dyspnea were the most frequent symptom indications for PS [22-24]. Although delirium was consistently the most common indication for PS in other studies, there was wide variability in its occurrence as an indicator (ranging from 13.8%-91.3%) [2]. In our practice, for patients with agitated delirium, the underlying cause was treated first with re-balancing water and electrolytes and provision of psychological support. Nevertheless, 39 patients required sedation for uncontrolled terminal stage agitation in our research. In this study, the frequency of pain as the indicator was lower than reported in previous researches [25-27], because PS was used for pain only after failure of interventions including opioids switching and escalation and other adjuvant drugs in our department.

In this study, Diazepam, a kind of benzodiazepine, was the drug used most often for sedation. It was the first choice because midazolam, the most popular drug for PS in Western practice, is not available in our department [28,29]. The second most used drug, haloperidol was not in the list of recommended drugs for PS according to the EAPC and NHAPC guidelines, but in many studies, it was still the first choice for patients with unmanageable agitated delirium. Haloperidol was used in 26% of sedated patients according to the review of 10 studies which enrolled 1807 patients combined [2]. Morphine was also not included in the list of the recommended drugs, but it may have produced an effect alongside sedation.

Continuous palliative sedation is increasingly accepted as a part of palliative care therapy in many countries [30]. Studies show that continuous sedation was performed in 2.5%-15% of all deaths in Europe [31-34]. 8.2% (20/244) patients in our study received continuous sedation. The decision to switch from intermittent to continuous sedation for these patients was based on the prevalence of uncontrolled symptoms. This indicated that continuous sedation can be acceptable for Chinese patients after sufficient discussion with family caregivers in some situations. As interfering with the dying process by causing patients to become unconscious, even with the intent to relieve physical suffering, was viewed as inhumane in Chinese traditional society, these results reflect to some extent a shift in end-of-life values.

The decision-making process also reflected the cultural values of each society. The reported degree of involvement of and information given to patients and families varies considerably [35,36]. Autonomy is one of the most valued rights in Western countries, especially in the United States, while in Eastern countries, illness is considered a family event rather than an individual occurrence [37]. Previous studies revealed that Asian patients prefer to leave end-of-life decisions to their family caregivers [38]. The family caregivers played a more important role in the decision-making process than patients. Liu JM’s research found that Chinese patients are less likely to sign their own do-not-resuscitate (DNR) orders [39]. The reason is that according to cultural tradition in China, the individual is viewed as embedded in the family and society. The decision making process for PS in our department is in accordance with this phenomenon. In our research, when patients were not conscious enough to make the decision for PS, we obtained informed consent only from their caregivers. Although to some extent, decisions from caregivers reflected the wishes of the patients, there was still potential for an ethical conflict when therapeutic decisions had to be made for these patients [35].

The key drawback of this study was that the retrospective research method had some limitations. Firstly, there were no standard form to record details of PS. Secondly, the information about the assessment of the symptom relief after receiving PS was insufficient to analyze. Another limitation is that as the data we gathered was in one cancer center, the result of this study cannot be extrapolated to other care settings in mainland China. To reduce the bias, studies involving multiple centers should be carried out in the future.

## Conclusion

To our knowledge, this research is the first study focused on the practice of PS for terminally ill cancer patients in mainland China. We recognized that PS is effective for reducing the suffering of these patients without hastening death. Prospective research is needed to determine the optimal conditions for Chinese patients including indications, decision making process, informed consent, cultural and ethical issues, type of sedation and drugs.
